# Chitin Conduits with Different Inner Diameters at Both Ends Combined with Dual Growth Factor Hydrogels Promote Nerve Transposition Repair in Rats

**DOI:** 10.3390/jfb14090442

**Published:** 2023-08-28

**Authors:** Fengshi Zhang, Bo Ma, Qicheng Li, Meng Zhang, Yuhui Kou

**Affiliations:** 1 Department of Orthopedics and Trauma, Peking University People’s Hospital, Beijing 100044, China; xmx066@pku.edu.cn (F.Z.); mengzh2008@bjmu.edu.cn (M.Z.); 2 Key Laboratory of Trauma and Neural Regeneration, Peking University, Beijing 100044, China; 3 National Center for Trauma Medicine, Beijing 100044, China

**Keywords:** peripheral nerve injury, nerve transposition, chitin, chitosan, nerve conduits, hydrogel, vascular endothelial growth factor, nerve growth factor

## Abstract

Severe peripheral nerve injuries, such as deficits over long distances or proximal nerve trunk injuries, pose complex reconstruction challenges that often result in unfavorable outcomes. Innovative techniques, such as nerve transposition repair with conduit suturing, can be employed to successfully treat severe peripheral nerve damage. However, cylindrical nerve guides are typically unsuitable for nerve transposition repair. Furthermore, angiogenic and neurotrophic factors are necessary to stimulate the emergence of axonal lateral sprouts, proximal growth, and the rehabilitation of neuron structures and functions. In the current study, we used chitosan to make chitin conduits with different inner diameters at both ends, combined with gelatin methacrylate hydrogels that can continuously release dual growth factors, namely, the vascular endothelial growth factor (VEGF) and the nerve growth factor (NGF), and evaluated its impact on nerve transposition repair in rats. At 16 weeks after the operation, our findings showed that the conduit combined with the dual growth factor hydrogel significantly improved the restoration of both motor and conduction functions of the nerve. In addition, histological analysis showed significant recovery of nerve fibers, target muscles, and neurons. In conclusion, the combination of chitin conduits with different inner diameters and dual growth factor hydrogels can significantly improve the effect of nerve transposition repair, which has important potential clinical value.

## 1. Introduction

Severe impairment and long-lasting disability can result from damage to the peripheral nerves [1,2]. Patients with severe peripheral nerve injuries, especially those with proximal nerve injuries such as brachial plexus avulsion and long-distance nerve defects, have a low chance of fully recovering their nerve function, resulting in limited functional recovery [3,4]. The extensive gap between the regenerated axons and the distant end-organs hinders the motor neurons’ capacity to regenerate axons into distant stumps. Extended denervation causes progressive atrophy of the peripheral nerve segments and associated muscles, leading to the inability to receive regenerating axons. Even when regenerating axons reach the neuromuscular junction, they cannot restore function due to the end-organs’ inability to accept reinnervation [5,6]. Therefore, optimal functional recovery requires nerve reinnervation within a shorter time after injury.

Peripheral nerve damage, including axon transection or endoneurium, perineurium, and epineurium rupture, elicits sprouting and regeneration of more lateral buds by the axons than by themselves. The outgrowing buds extend toward the distal stump and progress through the endoneurial tubes, culminating in the domination of the target organs. The proximal fibers produce a significantly larger number of lateral buds compared to the distal fibers and their endoneurial tubes, which is referred to as the nerve amplification effect [7,8]. This means that relatively thinner nerves can be used to repair relatively thicker damaged nerves and achieve a certain degree of functional recovery [9,10]. Hence, this repair method has been implemented in clinical treatment [11,12].

Large differences in diameter between the transposed nerves or the simultaneous repair of multiple distal nerves can cause problems during peripheral nerve damage repair. Traditional epineurial neurorrhaphy can lead to tension and even nerve suture failure at the suture site in these situations. In response to this issue, the researchers devised a reparative technique known as “conduit suturing.” This method involves suturing the nerve stumps to a conduit, eliminating the need for the direct suturing of the nerve stumps [13]. In the realm of neural tissue engineering, the creation of appropriate conduits for nerve restoration holds significant importance. A variety of compounds have been used in the preparation of nerve conduits, among these, numerous studies have investigated the use of chitosan and chitin [14,15]. Several research studies have indicated that chitosan and chitin could potentially engage with the microenvironment of nerve regeneration, leading to a decrease in the incidence of neuroma and an enhancement in axon regeneration [16,17]. Recently, chitosan has been utilized by our research group as a primary ingredient in the production of chitin conduits. These conduits have demonstrated exceptional biocompatibility and mechanical properties. Conduit suturing involves separately suturing the repaired nerve stumps via the conduit they are sleeved in, rather than directly suturing them [18]. However, even with the method of conduit suturing, if the conduit is a cylinder with the same diameter, the outer diameter of the nerve does not match the inner diameter of the conduit. To address this issue, using chitosan as a raw material, the conduit devised had varying diameters at both ends and was used to treat tibial nerve injuries via the proximal transposition of the common peroneal nerve in rats, resulting in tension-free suturing, nerve regeneration, and functional recovery [19]. However, further improvement is still necessary for the effectiveness of nerve transposition repair.

Various growth factors play a crucial role in the process of peripheral nerve regenerating [20,21]. Peripheral nerve regeneration and repair rely on two crucial factors, namely, the vascular endothelial growth factor (VEGF) and the nerve growth factor (NGF). VEGF, a powerful angiogenic factor, stimulates the development of fresh blood vessels, enhances blood circulation at the injury location, and supplies vital nourishment and oxygen for nerve regrowth [22]. NGF, belonging to the neurotrophic factor family, has the ability to improve the viability, growth, and differentiation of nerve cells, as well as stimulate the growth of axons [23]. Additionally, VEGF and NGF may have a potential synergistic effect in promoting nerve regeneration. VEGF promotes angiogenesis and increases blood flow, which is beneficial to supply oxygen and nutritional factors such as NGF to the injured site. NGF can enhance angiogenesis by upregulating the expression of VEGF [24]. VEGF and NGF can complement each other, leading to the promotion of nerve regeneration through their mutual support. Utilizing both VEGF and NGF in conjunction could offer a hopeful strategy for enhancing nerve regeneration and enhancing functionality. Previous studies have demonstrated that the combined application of VEGF and NGF promotes nerve damage repair [25]. However, whether the combined application of VEGF and NGF can promote the effect of nerve transposition repair remains unknown.

To ensure the lasting impact of growth factors in the injured region, it is imperative to guarantee their sustained efficacy when treating peripheral nerve injuries. Finding ways to consistently provide growth factors in the affected region is also a prominent concern in the study of peripheral nerve damage. Hydrogels are created by linking hydrophilic polymer chains together via chemical or physical means, resulting in three-dimensional networks. Their high-water content gives them structural and property similarities to biological tissue [26]. A wide range of natural and artificial polymers can be utilized to create hydrogels, which can be tailored to possess distinct characteristics such as compatibility with living organisms, the ability to breakdown naturally, and the controlled release of substances. Utilizing hydrogels as carriers for growth factors confers a notable advantage due to their ability to sustain the continuous release of these factors over a specific duration. The reason for this is that the hydrogel has the ability to soak up and preserve a significant quantity of water and growth factors within its composition. As the hydrogel breaks down, it slowly releases growth factors, which reduces the need for frequent application and minimizes any potential negative effects, ultimately enhancing both effectiveness and safety. Therefore, the unique properties of hydrogels make them an attractive option for the delivery of growth factors [27]. Gelatin methacrylate (GM) is a frequently utilized hydrogel substance that blends the qualities of both natural and synthetic biomaterials. They possess a three-dimensional framework that is conducive to the growth and differentiation of cells, along with remarkable biocompatibility and distinctive cellular response characteristics [28]. In addition, GM has good degradability and adjustable mechanical properties, the internal porous structure provides sufficient space for the delivery of growth factors, and its excellent biological properties can maintain the activity of growth factors during the delivery process, providing a basis for tissue repair [29]. GMs have been extensively utilized in the development of growth factor delivery systems due to the aforementioned benefits.

In this study, we prepared a gelatin methacrylate (GM) hydrogel capable of sustained release of the dual growth factors, namely, the vascular endothelial growth factor (VEGF) and the nerve growth factor (NGF), and its biological effects were preliminarily studied using in vitro experiments. Then, chitosan was used to make chitin conduits with different inner diameters at both ends, and the dual growth factor hydrogel was used in combination to evaluate its effect on nerve transposition repair in rats. Several techniques were employed to assess the effectiveness of the repair, such as the neurological function index, neuroelectrophysiological measurements, muscle wet weight, and the examination of nerve and muscle histology. This evaluation aimed to determine the success of the repair procedure.

## 2. Materials and Methods

### 2.1. Ethics Statement

The animal protocols and treatment followed the Guidelines for the Ethical Review of Experimental Animals for Animal Welfare, as specified by the Ethics Committee and Experimental Animal Center of Peking University People’s Hospital, Beijing, China. The study obtained an approved permit, with permit number 2022PHE050. Animal surgeries and care adhered to the guidelines set forth in the National Institutes of Health Guide for the Care and Use of Laboratory Animals (NIH Publication No. 85-23, revised 1985). The animal data were presented based on the ARRIVE 2.0 guidelines.

### 2.2. Construction of Hydrogel

The gelatin methacrylate (Engineering For Life, Suzhou, China) hydrogels were prepared following the instructions provided by the manufacturer. Briefly, the photoinitiator lithium phenyl-2,4,6-trimethylbenzoylphosphinate (LAP) was completely dissolved in the phosphate-buffered saline (PBS) (HyClone, Logan, UT, USA) in a water bath at 60 °C for 30 min. Then, the GM solid was completely dissolved in a LAP solution to form a 5% (*w*/*v*) uncrosslinked GM precursor solution. Then, the solution was irradiated with an ultraviolet light source (405 nm, 3 W; 20 s) to initiate the cross-linking of GM and turn the solution into hydrogel for subsequent experiments. For the preparation of the GM hydrogels loaded with growth factors, namely, GM/GF, VEGF (Novoprotein, Suzhou, China), and NGF (Novoprotein, Suzhou, China) were added to an uncrosslinked GM hydrogel solution at a concentration of 100 ng/mL and irradiated as described above.

### 2.3. Hydrogel Observation

The hydrogel morphology and pore structure were examined using a scanning electron microscope (SEM) (JSM-7900F, JEOL Ltd., Tokyo, Japan). The hydrogel was frozen solid in liquid nitrogen and subsequently subjected to freeze-drying using a vacuum freeze dryer (FDU-1100, EYALA, Tokyo, Japan). Next, a layer of gold/palladium (Au/Pd) was applied to the hydrogel surface to enable SEM observation.

### 2.4. Growth Factor Release Kinetics Profile

The detection of growth factor release kinetics in the hydrogel was performed using the enzyme-linked immunosorbent assay (ELISA) technique. Fifty microliters of hydrogels containing dual growth factors was submerged in 500 μL of PBS and positioned on a shaker at 60 rpm and 37 °C. The solutions were centrifuged at specified time points (1, 2, 3, 4, 5, 6, 7, 10, and 14 days) using a force of 1500× *g* for a duration of 5 min. Supernatants were collected for ELISA, and 500 μL of PBS was added. The growth factor concentrations in the supernatants were quantified using an ELISA kit (Fine Biotech, Wuhan, China) according to the manufacturer’s instructions. The release kinetics were calculated using the following formula: m_t_ = m_1_ + m_2_ + m_3_ + …… + m_x_, where m_t_ refers to the cumulative amount of the released growth factor, and m_x_ indicates the released amount measured on day x.

### 2.5. Cell Culture

HUVEC (CRL-1730) and PC-12 (CRL-1721) cells were acquired from the American Type Culture Collection (Manassas, VA, USA). The HUVECs were cultured in Dulbecco’s modified Eagle’s medium (DMEM) (Gibco, Life Technologies, Carlsbad, CA, USA) supplemented with 10% fetal bovine serum (FBS) (Gibco, Life Technologies, Carlsbad, CA, USA). PC-12 cells were cultured in a RPMI-1640 medium (Gibco, Life Technologies, Carlsbad, CA, USA) supplemented with 5% FBS (Gibco, Life Technologies, Carlsbad, CA, USA) and 10% horse serum (Gibco, Life Technologies, Carlsbad, CA, USA). All cells were cultured in a humidified incubator (Thermo Fisher Scientific, Waltham, MA USA) with 95% air and 5% CO_2_ at 37 °C.

### 2.6. Cell Proliferation Assay

By establishing a hydrogen peroxide-induced oxidative stress injury model in HUVECs, the effect of the double growth factor hydrogel on cell proliferation was observed. For each group, 1 × 10^5^ HUVECs were seeded in 12-well plates. In the control group, the cells were incubated with DMEM containing 5% FBS. In the injury group, hydrogen peroxide was added. In the injury/GM group and injury/GM/GF group, except for hydrogen peroxide, the leachates of GM and GM/GF were added separately.

HUVEC proliferation was evaluated using a 5-ethynyl-2′-deoxyuridine (EdU) assay. The cells were labeled by adding EdU reagent (Beyotime, Shanghai, China) and incubating for a duration of 2 h. Following PBS washing, the cells were treated with 4% paraformaldehyde (Solarbio, Beijing China) for 20 min, permeabilized using 0.5% Triton X-100 (Solarbio, Beijing China) for 10 min, and subsequently incubated with the click-reaction reagent in the dark at room temperature for 30 min. Nuclei were stained using Hoechst 33342 reagent (Beyotime, Shanghai, China). The staining results were observed via a 3D fluorescence imaging system (HOOKE INSTRUMENTS, Changchun, China). The nuclei of proliferating cells have red fluorescence, and the nuclei of all cells have blue fluorescence. The HUVEC proliferation rate was calculated as follows: proliferation rate (%) = N_r_/N_b_ × 100, where N_r_ represents the number of nuclei with red fluorescence, and N_b_ represents the number of nuclei with blue fluorescence.

### 2.7. Tube Formation Assay

For each group, 3 × 10^4^ HUVECs were suspended in 200 μL of different mediums and then plated on Matrigel (Corning Inc., Corning, NY, USA) in 48-well plates. In the control group, the cells were suspended in DMEM containing 5% FBS. In the GM group and GM/GF group, the leachates of GM and GM/GF were added separately. Tube formation was evaluated after incubation for 4 h.

### 2.8. Cell Migration Assay

*HUVECs* (2 × 10^5^) were seeded in 12-well plates and incubated until confluent. A sterile 200 µL pipette tip was utilized to gently scrape the cell layer’s surface, followed by rinsing with PBS to eliminate any cells that have become detached. In the control group, the cells were incubated with DMEM containing 2% FBS. In the GM group and GM/GF group, the leachates of GM and GM/GF were added separately. The cells were observed and photographed using a microscope (HOOKE INSTRUMENTS, Changchun, China) after 12 and 18 h. The HUVEC migration rate was calculated as follows: migration rate (%) = (A_0_ − A_x_)/A_0_ × 100, where A_0_ represents the initial wound area, and A_x_ represents the wound area in the x hour.

### 2.9. Apoptosis Detection

By establishing a hydrogen peroxide-induced oxidative stress injury model in PC-12 cells, the effect of the double growth factor hydrogel on cell apoptosis was observed. For each group, 5 × 10^4^ PC-12 cells were seeded in 12-well plates. In the control group, the cells were incubated with an RPMI-1640 medium containing 5% FBS and 10% horse serum. In the injury group, hydrogen peroxide was added. In the injury/GM group and injury/GM/GF group, except for hydrogen peroxide, the leachates of GM and GM/GF were added separately.

PC-12 cell apoptosis was detected using a TdT-mediated dUTP nick-end labeling (TUNEL) apoptosis assay. Following a gentle wash with PBS, the cells were fixed in 4% paraformaldehyde (Solarbio, Beijing China) for 20 min. Subsequently, they were permeabilized with 0. 5% Triton X-100 (Solarbio, Beijing China) for a period of 10 min. Following a gentle wash with PBS, a TUNEL detection solution (Beyotime, Shanghai, China) was added and incubated at 37 °C for 60 min in the dark. Nuclei were stained using 2-(4-amidinophenyl)-6-indolecarbamidine dihydrochloride (DAPI) (Beyotime, Shanghai, China). The staining results were observed via a 3D fluorescence imaging system (HOOKE INSTRUMENTS, Changchun, China). The nuclei of apoptotic cells have green fluorescence, and the nuclei of all cells have blue fluorescence. The PC-12 cell apoptosis rate was calculated as follows: apoptosis rate (%) = N_g_/N_b_ × 100, where N_g_ represents the number of nuclei with green fluorescence, and N_b_ represents the number of nuclei with blue fluorescence.

### 2.10. Fabrication of Chitin Conduits with Different Inner Diameters at Both Ends

The manufacturing mold used for the conduit is made entirely of stainless steel. The mold consists of two cylindrical guiding rods with varying diameters that are connected by a conical piece. The diameter of the thinner guiding rod is 0.8 mm, and the diameter of the thicker guiding rod is 1.6 mm. The conical piece’s end diameters are in alignment with the diameter of the guiding rods on both sides. The guiding rod diameter is crucial in shaping the conduit, as it determines the final output size and shape.

**Figure 1:**
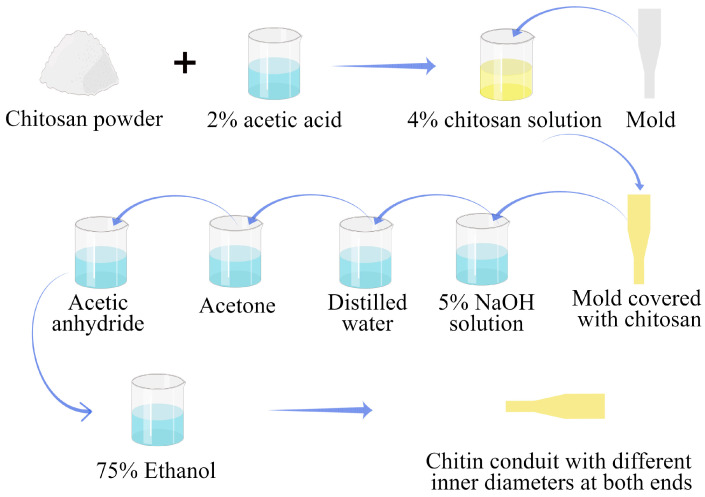
The preparation process of chitin conduits with different inner diameters at both ends.

The chitin conduits were constructed using previously described methods [30]. To prepare a 4% (*w*/*v*) chitosan solution, chitosan (Beijing Chemical Factory, Beijing, China) with a deacetylation level exceeding 70% and a molecular weight ranging from 15–50 × 10^4^ Da was dissolved in an aqueous solution of acetic acid with a concentration of 2%. The bubbles in the solution were removed using a vacuum pump. The specific mold was slowly immersed in the chitosan solution so that the surface of the mold was evenly covered with the chitosan solution and then immersed in an NaOH aqueous solution to solidify the chitosan solution on the mold surface. After the solution solidified, distilled water was used to wash the residual NaOH, and then acetone was used to dehydrate the chitosan. Next, chitosan was acetylated with acetic anhydride to form chitin conduits with different inner diameters at both ends. The conduits were then gently peeled from the mold and stored in a 75% ethanol solution for subsequent use (Figure 1). The conduit was labeled CT.

### 2.11. Construction of the Conduits with Hydrogels

The uncrosslinked GM hydrogel precursor solution was filled into the conduits with a syringe. To create the hydrogel, the solution was exposed to a 405 nm, 3 W ultraviolet light source for 20 s, resulting in crosslinking. This conduit was labeled CT/GM. The uncrosslinked GM/GF hydrogel precursor solution loaded with VEGF and NGF was filled into the conduit and irradiated with the same method. This conduit was labeled CT/GM/GF.

### 2.12. Experimental Animal Preparation

We purchased thirty-six female specific-pathogen free Sprague-Dawley rats from Beijing Vital River Laboratory Animal Technology Co., Ltd., Beijing, China (license No. SCXK (Jing) 2021-0006). These rats were six weeks old, weighed between 200–220 g and were housed in the Experimental Animal Center of Peking University People’s Hospital. The rats were housed in a controlled environment maintained at a temperature of 24 °C, with a relative humidity of 50–55% and a light/dark cycle of 12 h each. The rats were given unrestricted access to regular pellet food and fresh water. The rats were randomly divided into four groups, with nine rats in each group. The groups were named as follows: sham group, CT group (common peroneal nerve repairing the tibial nerve using CT conduit), CT/GM group (common peroneal nerve repairing the tibial nerve using CT/GM conduit), and CT/GM/GF group (common peroneal nerve repairing the tibial nerve using CT/GM/GF conduit). All measures were taken to reduce animal suffering, and the study followed ethical protocols.

### 2.13. Nerve Injury and Repair Model

Following the administration of 2.5% isoflurane, the experimental animals were anesthetized, and the hair on the lower limb’s right side was removed. To uncover the right sciatic nerve and its two main divisions, namely, the common peroneal nerve and tibial nerve, surgical operations were conducted. All surgical procedures were performed under a surgical microscope and aseptic conditions using standard microsurgical techniques. To reduce tension on the repair site, a technique involving the splitting of the dorsal gluteal region was employed following careful dissection of the nerve branches.

**Figure 2:**
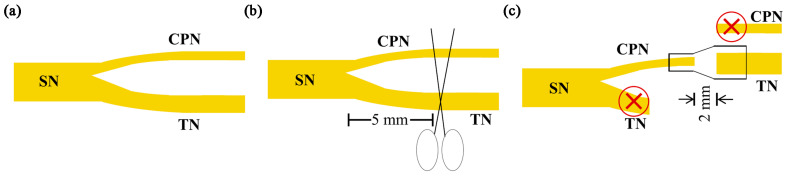
Schematic diagram of surgical procedures. (**a**) The sciatic nerve and its two main branches, namely, the common peroneal nerve and tibial nerve. (**b**) The tibial nerve and the common peroneal nerve were cut 5 mm away from the point where they split. (**c**) The nearby end of the common peroneal nerve was utilized for the restoration of the far end of the tibial nerve. CPN: common peroneal nerve; SN: sciatic nerve; TN: tibial nerve.

The common peroneal nerve and tibial nerve were cut five millimeters distal to the bifurcation in the CT group, CT/GM group, and CT/GM/GF group. The nearby ends of the tibial nerve and the distant ends of the common peroneal nerve were tied with a 10-0 nylon thread, and the tied ends were stitched to the surrounding muscles. The tibial nerve’s distal stump was repaired by utilizing the nearby end of the common peroneal nerve. Approximately 2 mm into the thinner end of the conduit, the proximal stump of the common peroneal nerve was inserted. Approximately 2 mm, the thicker end of the conduit received the insertion of the tibial nerve’s distal stump. A 2 mm space was maintained between the proximal and distal stumps, which were then secured to the conduit using a 10-0 nylon suture. In the end, the surgical area was closed using 4-0 nylon sutures in layers (Figure 2).

In the sham group, we performed an incision and exposure of the sciatic nerve and its branches without inducing harm to the nerve fibers. The surgical area was closed using 4-0 nylon sutures in layers.

### 2.14. General and Local Conditions of Rats after Operation

Following the surgery, the rats were provided with regular nourishment while their daily progress was meticulously documented. This encompassed their diet, physical activity, healing of surgical wounds, and movement of their right hind limbs. After a period of 16 weeks following the surgery, the tibial nerve that had been repaired was uncovered, and an examination was conducted to observe the structure of the nerve as well as the presence of any adhesion in the surrounding tissue.

### 2.15. Neurological Function Index

The Rat and Mouse Gait Analysis Processing System v1. 09 (Zhongshi Dichuang Technology, Beijing, China) was utilized to assess the restoration of nerve motor function 16 weeks postsurgery. Every single rat traversed a sealed passage made up of a transparent floor and walls made of dark plastic. The tibial functional index (TFI) was determined using the provided formula.

$$TFI=-\frac{37.2\left(EPL-NPL\right)}{NPL}+\frac{104.4(ETS-NTS)}{NTS}+\frac{45.6\left(EITS-NITS\right)}{NITS}-8.8$$

EPL represents the measurement from the heel to the highest point of the third toe on the right hind limb, while NPL represents the distance from the heel to the top of the third toe on the left hind limb. ETS refers to the distance between the first and fifth toes on the right hind limb, and NTS refers to the distance between the first and fifth toes on the left hind limb. Additionally, EITS represents the measurement between the second and fourth toe on the right hind limb, and NITS represents the measurement between the second and fourth toe on the left hind limb.

### 2.16. Nerve Electrophysiological Detection

After 16 weeks of surgery, a MedlecSynergy electrophysiological device (04oc003, Oxford Instrument Inc., Abingdon, UK) was used to assess the recovery of regenerated nerve conduction on the right side in every rat. Under 2.5% isoflurane anesthesia, the tibial nerve on the right side and the gastrocnemius muscle were carefully uncovered. Stimulation occurred at both the near and far ends of the tibial nerve, while recording took place in the center of the gastrocnemius muscle. Recordings were made of the amplitude and latency of the compound muscle action potential (CMAP), and subsequently, the nerve conduction velocity was determined using the prescribed method. The conduction velocity of the tibial nerve was determined by calculating the ratio of the length of the nerve trunk (dl) to the difference in action potential latency (dt) between proximal and distal tibial nerve stimulation.

### 2.17. Immunofluorescence Staining of Regenerated Nerve Fibers

At 16 weeks after surgery, the tibial nerve was harvested after carbon dioxide euthanasia. The nerve was treated with 4% paraformaldehyde for a duration of 24 h, followed by slicing the nerve into transverse sections of 12 μm thickness using a frozen slicer. Immunofluorescence staining was conducted using an established protocol with NF200 (MilliporeSigma, Burlington, MA, USA) staining targeted toward monitoring axonal regeneration and S100 (MilliporeSigma, Burlington, MA, USA) staining to detect Schwann cells. In short, sections of nerve tissue were incubated with primary antibodies that targeted NF200 and S100 for an overnight period at 4 °C, followed by rinsing with PBS. Afterwards, secondary antibodies conjugated with Alexa488 (Abcam, Cambridge, UK) and Alexa594 (Abcam, Cambridge, UK) were added and incubated at room temperature for 1 h. DAPI (Beyotime, Shanghai, China) was applied to the nucleus and incubated for 5 min. The tissue sections were observed via a 3D fluorescence imaging system (HOOKE INSTRUMENTS, Changchun, China).

### 2.18. Morphological Evaluation of Regenerated Nerve Fibers

After a period of 16 weeks following the surgery, the tibial nerve was extracted upon administering carbon dioxide euthanasia. The nerve tissue was fixed with 2.5% glutaraldehyde for 24 h, followed by staining with 1% osmic acid (Electron Microscopy Sciences, Hatfield, PA, USA). To prepare the sample, a gradient concentration of acetone was utilized for dehydration. Afterwards, the specimens were encased in epoxy resin and then sectioned into semithin slices measuring 700 nm in thickness and ultrathin slices measuring 70 nm in thickness. Semithin sections were stained using a 1% solution of toluidine blue (Solarbio, Beijing, China) and examined with an optical microscope (Olympus Corporation, Tokyo, Japan) to calculate the density of the nerve fibers. The ultrathin sections were treated with uranyl acetate and lead citrate (Electron Microscopy Sciences, Hatfield, PA, USA) and examined using a transmission electron microscope (Olympus Corporation, Tokyo, Japan). ImageJ software (version 1.8.0, LOCI, University of Wisconsin, Madison, WI, USA) was utilized to measure the diameters of the axons and nerve fibers. The formula used to calculate myelin thickness is (fiber diameter minus axon diameter) divided by 2.

### 2.19. Target Muscle Recovery

After a period of 16 weeks following the surgical procedure, the gastrocnemius muscles were gathered from both sides, and their weight was measured. The wet weight ratio of the gastrocnemius muscles was calculated by dividing the wet weight of the muscle on the right side by the wet weight of the muscle on the left side. Next, the muscles on the right side were immobilized in 4% paraformaldehyde at a temperature of 4 °C for the entire night. Afterwards, they were embedded in paraffin and sliced into 6 µm thick sections. Afterwards, Masson’s trichrome stain was applied to the sections, and random images of the muscle cross-section were taken from five fields in each image. ImageJ Software (version 1.8.0, LOCI, University of Wisconsin, Madison, WI, USA) was used to measure the cross-sectional area of the muscle fibers in these images. Next, the wet weight proportion and average cross-sectional area of the gastrocnemius muscle fibers on the right side were computed and compared across the four groups.

### 2.20. Fluor-Gold (FG) Retrograde Tracing

After a period of sixteen weeks following the surgical procedure, the nerves of the rats were revealed through the initial cut while under the influence of 2.5% isoflurane anesthesia. The proximal end of the common peroneal nerve was injected with a 4% FG (Fluorochrome, Denver, CO, USA) solution, after which the incision was sutured. Following a week, the rats were immobilized using a solution of 0.9% NaCl and 4% paraformaldehyde via transcardiac perfusion while under anesthesia. The L4–L6 spinal cord and dorsal root ganglia (DRG) were then removed, fixed with a 4% paraformaldehyde solution at 4 °C for 24 h, and subsequently dehydrated using gradient concentrations of sucrose solutions (from 10% to 20% to 30%). The specimens were embedded in the OCT complex and then cut serially into sections. The spinal cord samples were cut into sections that were 25 µm thick, while the DRG samples were cut into sections that were 20 µm thick. To assess the quantity of motor neurons in the spinal cord and sensory neurons in the DRG after regeneration, the sections were examined with a 3D fluorescence imaging system (Hooke Instruments, Changchun, China).

### 2.21. Statistical Analysis

The study employed a blind method for both image analysis and behavior assessments. The mean and standard deviation (SD) were used to present the findings. SPSS 22 was utilized to conduct statistical analysis. Multiple groups were compared using the one-way analysis of variance (ANOVA). If there was notable variation between groups, Tukey’s post hoc test was utilized to conduct pairwise comparisons. A *p* value of less than 0.05 was chosen as the level of significance.

## 3. Results

### 3.1. Synthesis and Characterization of Hydrogels

**Figure 3:**
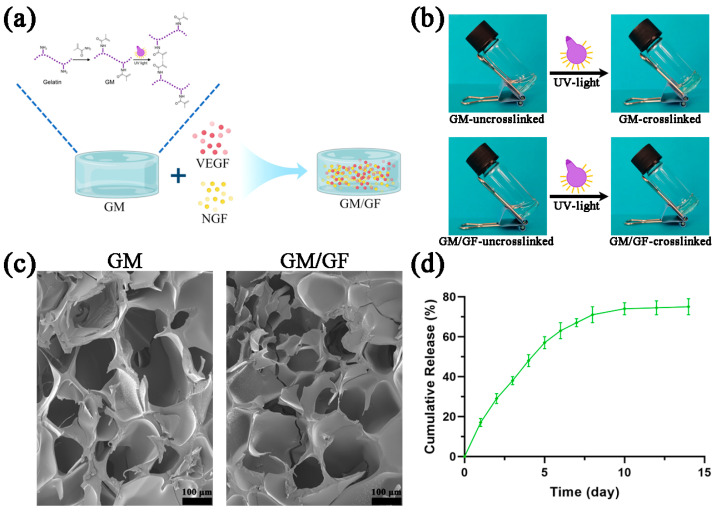
Synthesis and characterization of hydrogels. (**a**) Schematic diagram of the preparation of hydrogels loaded with VEGF and NGF. (**b**) Gross view of hydrogels before and after crosslinking. (**c**) SEM images of GM and GM/GF hydrogels. (**d**) Cumulative release profile of the GM/GF hydrogel. Data are presented as the mean ± standard deviation (SD).

Figure 3 shows the synthesis process and characterization of the hydrogels. Figure 3a shows a schematic diagram of the preparation of hydrogels loaded with VEGF and NGF. Figure 3b shows the gross view of hydrogels before and after crosslinking. The uncrosslinked GM and GM/GF were in solution form. When UV light was used for crosslinking, the solution became a hydrogel. SEM was used to observe the microstructure of GM and GM/GF hydrogels. As shown in Figure 3c, both GM and GM/GF hydrogels had porous structures, which were conducive to cell adhesion and material exchange. As shown in Figure 3d, the growth factors were continuously and stably released from the GM/GF hydrogel, suggesting that GM/GF can continuously exert biological effects in vitro and in vivo.

### 3.2. Dual Growth Factor Hydrogel Promotes HUVEC Proliferation, Tube Formation, Migration

**Figure 4:**
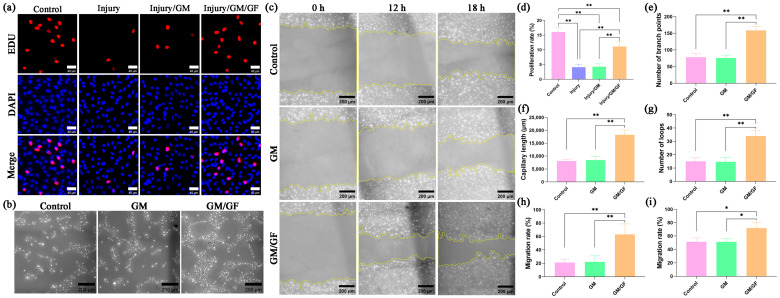
HUVEC proliferation, tube formation, and migration. (**a**) EdU staining images of HUVECs. (**b**) Tube formation images of HUVECs. (**c**) Migration images of HUVECs. (**d**) Statistical analysis of the proliferation rate. (**e**) Statistical analysis of the number of branch points. (**f**) Statistical analysis of capillary length. (**g**) Statistical analysis of the number of loops. (**h**) Statistical analysis of the migration rate at 12 h. (**i**) Statistical analysis of the migration rate at 18 h. Data are presented as the mean ± SD. * *p* < 0.05; ** *p* < 0.01.

The EdU staining method was employed to evaluate HUVEC proliferation in each group. The results were analyzed to determine the cell proliferation rate. Figure 4a displays representative EDU staining images for each group. The results indicated that the injury, injury/GM, and injury/GM/GF groups had a significantly lower proliferation rate than the control group (*p* < 0.01). Nevertheless, the rate of proliferation in the injury/GM/GF group exhibited a considerably greater increase than that in both the injury group and injury/GM group (*p* < 0.01). No notable distinction was observed between the injury group and the injury/GM group (Figure 4d).

To assess the tube formation of HUVECs in each group, a tube formation assay was utilized. For each group, Figure 4b shows representative tube formation images. The GM/GF group exhibited a considerably greater number of branch points, capillary length, and loops than the control and GM groups, as depicted in Figure 4e–g (*p* < 0.01). Nevertheless, there was no notable distinction observed between the control group and the GM group.

To assess the migration of HUVECs in each group, a wound healing assay was utilized. Representative migration images for each group are shown in Figure 4c. The migration rate in the GM/GF group at 12 h and 18 h, as depicted in Figure 4h,i, exhibited a significant increase compared to the control and GM groups (*p* < 0.05 or *p* < 0.01). Nevertheless, there was no notable distinction observed between the control group and the GM group.

### 3.3. Dual Growth Factor Hydrogel Alleviates PC-12 Apoptosis

**Figure 5:**
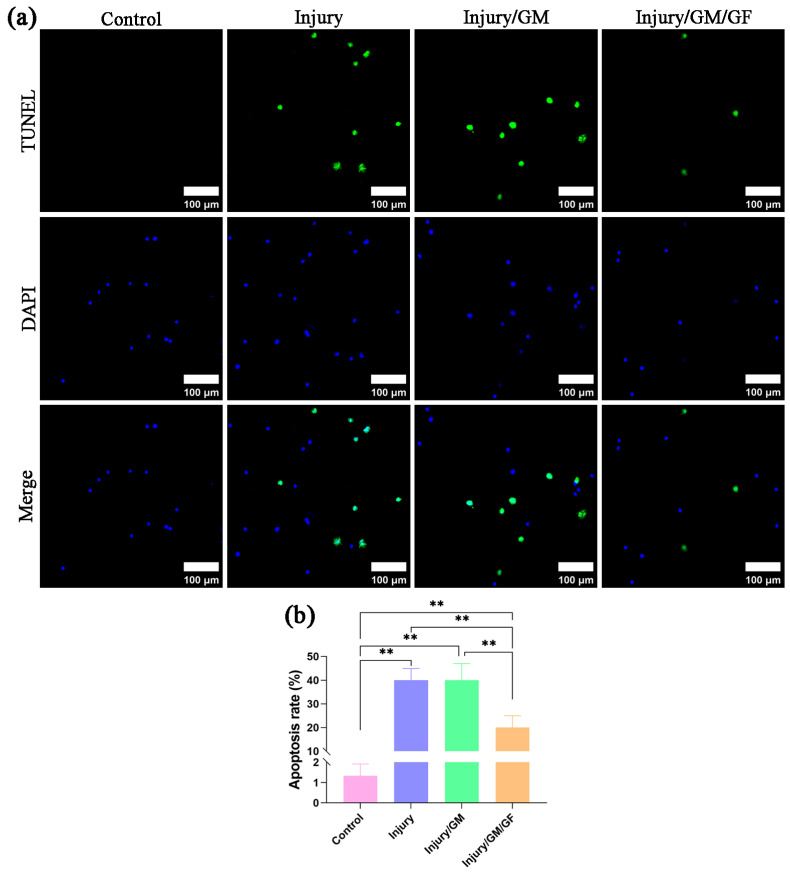
Apoptosis rate of PC-12 cells. (**a**) TUNEL staining images of PC-12 cells. (**b**) Statistical analysis of the apoptosis rate. Data are presented as the mean ± SD. ** *p* < 0.01.

Figure 5 displays the outcomes of TUNEL staining. Representative images of TUNEL staining for each group are shown in Figure 5a. As depicted in Figure 5b, the findings showed that the injury, injury/GM, and injury/GM/GF groups exhibited a markedly elevated rate of apoptosis in comparison to the control group (*p* < 0.01). Nevertheless, the rate of apoptosis in the injury/GM/GF group exhibited a notable decrease compared to both the injury group and injury/GM group (*p* < 0.01). The results showed no notable distinction between the injury group and the injury/GM group.

### 3.4. Chitin Conduits Combined with Dual Growth Factor Hydrogels Improve Nerve Conduction, Relieve Target Muscle Atrophy, and Promote Nerve Function Recovery

**Figure 6:**
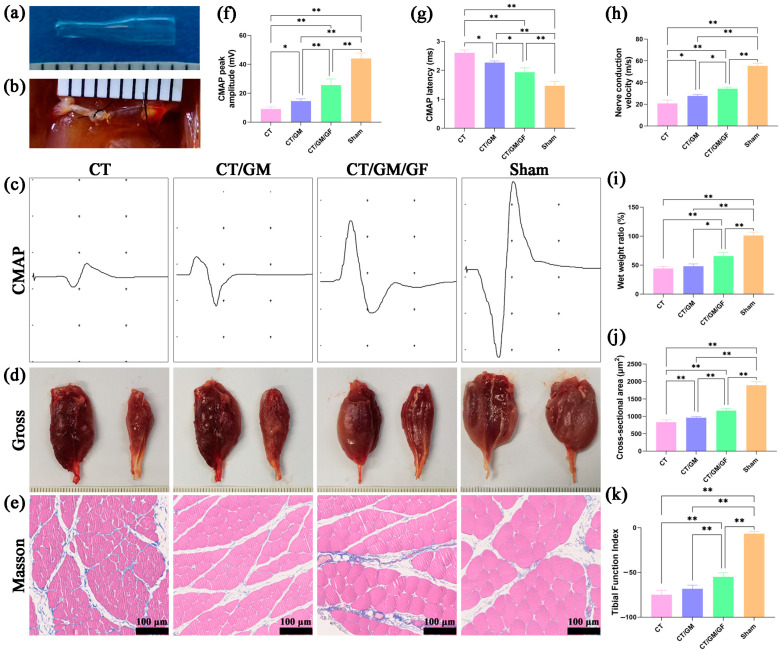
Chitin conduit, nerve conduction, target muscle, and nerve function. (**a**) Gross view of the chitin conduit with different inner diameters at both ends. (**b**) Representative photograph of nerve transposition repair using conduits with different inner diameters at both ends. (**c**) Representative CMAP waveform in each group. (**d**) Representative images of normal (left) and injured (right) gastrocnemius muscles. (**e**) Representative images of Masson staining of the right gastrocnemius muscle in each group. (**f**) Statistical analysis of CMAP peak amplitude. (**g**) Statistical analysis of CMAP latency. (**h**) Statistical analysis of nerve conduction velocity. (**i**) Statistical analysis of the wet weight ratio in each group. (**j**) Statistical analysis of the mean muscle fiber cross-sectional area in each group. (**k**) The TFI in each group. In (**a**,**b**,**d**) the distance between two adjacent black lines is 1 mm. Data are presented as the mean ± standard deviation (SD). * *p* < 0.05; ** *p* < 0.01.

According to Figure 6a, the conduit has varying inner diameters at its two ends. The thinner end has an inner diameter of 0.8 mm, while the thicker end has an inner diameter of 1.6 mm. A conical structure connects the thinner end and thicker end. The thickness of the wall was approximately 0.3 mm. The conduit has an overall transparent appearance. According to Figure 6b, the conduit’s inner diameter corresponds to the nerves’ diameter.

During the 16th week following the surgical procedure, rats from every group were subjected to electrophysiological examinations to determine the restoration of nerve conduction. The amplitude of CMAP is directly related to the quantity of muscle fibers that are innervated, while the latency of CMAP is inversely related to the thickness of the myelin sheath surrounding the nerve fibers. Figure 6c demonstrates typical CMAP waveforms for each rat group. Figure 6f shows that the CT/GM and CT/GM/GF groups exhibited considerably reduced CMAP peak amplitudes in comparison to the sham group (*p* < 0.01), while these groups demonstrated significantly higher amplitudes when compared to the CT group (*p* < 0.05 or *p* < 0.01). Furthermore, the magnitude in the CT/GM/GF group was considerably greater than that in the CT/GM group (*p* < 0.01). Moreover, as shown in Figure 6g, the CMAP latency of the CT/GM and CT/GM/GF groups was significantly higher than that of the sham group (*p* < 0.01) but significantly lower than that of the CT group (*p* < 0.05 or *p* < 0.01). The latency in the CT/GM/GF group was considerably less than that in the CT/GM group (*p* < 0.05). Figure 6h shows that the nerve conduction velocity was considerably decreased in both the CT/GM and CT/GM/GF groups compared to the sham group (*p* < 0.01), but it was notably higher than in the CT group (*p* < 0.05 or *p* < 0.01). The velocity in the CT/GM/GF category was considerably greater than that in the CT/GM group (*p* < 0.05).

At 16 weeks following the surgical procedure, both gastrocnemius muscles were obtained from rats, weighed, photographed, and subjected to Masson staining specifically on the right side. Representative images of the left and right gastrocnemius muscles for each group can be seen in Figure 6d, whereas Figure 6e displays the Masson staining outcomes of the right gastrocnemius muscle across various groups. In Figure 6i, it was observed that the wet weight ratio of the right and left gastrocnemius muscles in the CT, CT/GM, and CT/GM/GF groups exhibited a significant decrease when compared to the sham group (*p* < 0.01). The ratio in the CT/GM/GF group was considerably greater than that in the CT group and CT/GM group (*p* < 0.05 or *p* < 0.01). Nevertheless, there was no notable distinction observed between the CT group and the CT/GM group. Moreover, as shown in Figure 6j, the mean muscle fiber cross-sectional area in the CT/GM and CT/GM/GF groups was considerably smaller than that in the sham group (*p* < 0.01) but notably larger than that in the CT group (*p* < 0.01). The CT/GM/GF group had a considerably larger area than the CT/GM group (*p* < 0.01).

At 16 weeks postsurgery, a functional index analysis was conducted on each group to evaluate the recovery of motor function. The TFI of each group is displayed in Figure 6k. The TFI of the CT, CT/GM, and CT/GM/GF groups exhibited a significant decrease compared to that of the sham group (*p* < 0.01). The TFI in the CT/GM/GF group was considerably greater than that in the CT group and CT/GM group (*p* < 0.01). Nevertheless, there was no notable distinction observed between the CT group and the CT/GM group.

### 3.5. Chitin Conduits Combined with Dual Growth Factor Hydrogels Improve Axonal Regeneration and Recovery of Neurons

**Figure 7:**
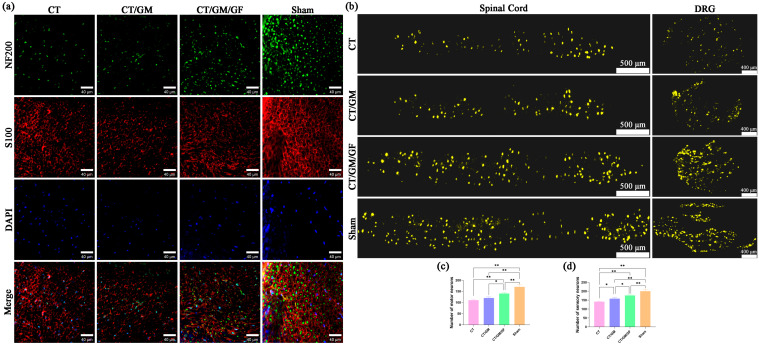
Immunofluorescence staining of regenerated nerve tissue and FG retrograde tracing. (**a**) Representative images of immunofluorescence staining of regenerated nerve tissue. Green fluorescence serves as an indicator of regenerating axons, red fluorescence represents Schwann cells, and blue fluorescence reveals the presence of nuclei. (**b**) Representative images of FG-labeled neurons in each group. (**c**) Statistical analysis of the number of FG-labeled motor neurons in each group. (**d**) Statistical analysis of the number of FG-labeled sensory neurons in each group. Data are presented as the mean ± SD. * *p* < 0.05; **p < 0.01.

Immunofluorescence staining was conducted on the cross-sections of the regenerated nerves of rats in every group at the 16th week following nerve surgery, and the findings are illustrated in Figure 7a. The presence of regenerating axons is indicated by green fluorescence, while Schwann cells are represented by red fluorescence, and the presence of nuclei is revealed by blue fluorescence. According to the findings, the CT/GM/GF group exhibited a higher regeneration of axons than both the CT/GM group and CT group.

The number of motor neurons in the anterior horn of the spinal cord and sensory neurons in the DRG of each group were evaluated using the FG retrograde tracing method after a 16-week postoperative period. The analysis of the results was conducted to ascertain the recuperation of nerve cells in every group. The observation of FG-labeled motor neurons and sensory neurons for each group is depicted in Figure 7b. As shown in Figure 7c, the quantity of motor neurons labeled with FG in the CT, CT/GM, and CT/GM/GF groups exhibited a significant decrease compared to that in the sham group (*p* < 0.01). The CT/GM/GF group exhibited a substantially greater number than the CT group and CT/GM group (*p* < 0.05 or *p* < 0.01). Nevertheless, there was no notable distinction observed between the CT group and the CT/GM group. As shown in Figure 7d, the quantity of sensory neurons labeled with FG in the CT/GM and CT/GM/GF groups exhibited a significant decrease compared to that in the sham group (*p* < 0.01), yet it was notably higher than that in the CT group (*p* < 0.05 or *p* < 0.01). The CT/GM/GF group exhibited a substantially greater number than the CT/GM group (*p* < 0.05).

### 3.6. Chitin Conduits Combined with Dual Growth Factor Hydrogels Improve Morphological Recovery of Regenerated Nerve Fibers

**Figure 8:**
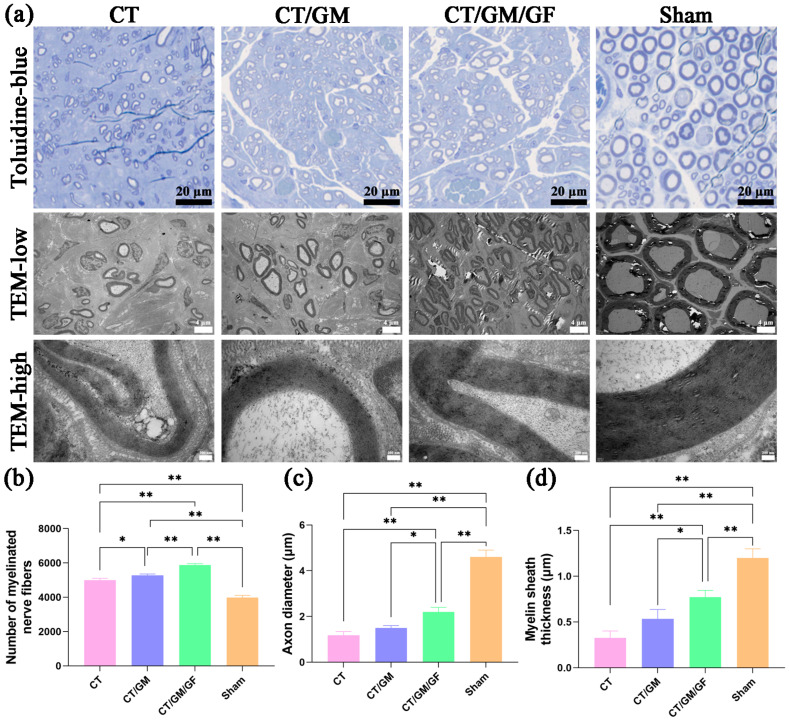
Evaluation of the histological and morphological characteristics of the regenerated nerve fibers 16 weeks postsurgery. (**a**) Representative images of toluidine blue staining and transmission electron microscopy for each group. (**b**) Statistical analysis of the quantity of regenerated axons within each group. (**c**) Statistical analysis of the axon diameter of the regenerated nerves in each group. (**d**) Statistical analysis of the thickness of the myelin sheath in the regenerated nerves within every group. Data are presented as the mean ± SD. * *p* < 0.05; ** *p* < 0.01.

Figure 8 displays the findings from the examination of the regenerated rat nerve cross-section using toluidine blue staining and electron microscopy. Representative images of toluidine blue staining and transmission electron microscopy for each group are shown in Figure 8a. The uniformity of regenerated myelinated nerve fibers in the CT, CT/GM, and CT/GM/GF groups was lower than that in the sham group. According to the findings, the CT/GM and CT/GM/GF groups exhibited a markedly greater quantity of myelinated nerve fibers than the CT and sham groups (*p* < 0.05 or *p* < 0.01). Additionally, the CT/GM/GF group exhibited a substantially greater number than the CT/GM group (*p* < 0.01) (Figure 8b). Figure 8c depicts the diameter of the regenerated nerve axons in each group. The axon diameter was significantly lower in the CT, CT/GM, and CT/GM/GF groups than in the other groups (*p* < 0.01). The CT/GM/GF group exhibited a significantly higher diameter than the CT group and CT/GM group (*p* < 0.05 or *p* < 0.01). Nevertheless, there was no notable distinction observed between the CT group and the CT/GM group.

Figure 8d displays the thickness of the myelin sheath in the regenerated nerves. The thickness was significantly lower in the CT, CT/GM, and CT/GM/GF groups than in the other groups (*p* < 0.01). The CT/GM/GF group exhibited a significantly higher thickness than the CT group and CT/GM group (*p* < 0.05 or *p* < 0.01). Nevertheless, there was no notable distinction observed between the CT group and the CT/GM group.

## 4. Discussion

Different degrees of severity exist in peripheral nerve injuries, with long-distance nerve deficits and proximal nerve trunk injuries being especially severe [31,32]. In this case, the achievement of long-distance axonal regeneration for reinnervating target organs is challenging. During this process, the target organs may undergo degeneration [33]. If the target organ is already degraded, even if the regenerating axon grows into it, the repair is still less effective [34]. Therefore, successful repair of such severe peripheral nerve injury, namely, long-distance nerve deficits and proximal nerve trunk injuries, involves shortening the denervation time of the target organ as much as possible.

In previous studies, we observed that there is an amplification effect during peripheral nerve regeneration [7,18]. During the initial phases of nerve recovery after an injury, a proximal nerve fiber develops numerous side branches and stretches out toward the distal endoneural tubes in an effort to enter them [35,36]. When there are more distal endoneural tubes than proximal nerve axons, there is sufficient room for regenerated axons to develop, resulting in an enhancement of the nerve fibers [37,38]. The amplification effect could potentially be utilized as an innovative approach in the medical management of severe peripheral nerve damage. Thinner proximal nerves can be utilized to repair thicker distal damaged nerves. Nerve transposition repair can reduce the denervation of the target organ and promote cost-effective rehabilitation by allowing the donor nerve to generate additional sprouts toward the distal stump, thereby restoring the structure and function of the damaged nerve [39,40]. The distal tibial nerve was repaired using the common peroneal nerve as a donor in this research. The findings demonstrated partial restoration of the distal tibial nerve function, which is consistent with the amplification effect of nerve regeneration.

In recent years, researchers have developed a variety of nerve transposition repair techniques. For example, brachial plexus nerve injury was repaired using the ipsilateral or contralateral cervical 7 nerves, parasympathetic nerves, and phrenic nerves [4]; median nerve injury was repaired using the musculocutaneous nerves [13]; ulnar nerve injury was repaired using the spinoglossus branch [41], and so on. In nerve transposition repair, the proximal ends of these particular nerves need to be sutured to the distal ends of the other nerves. Unlike the common suturing method used to treat nerve injuries, the diameters of the proximal and distal nerves are often not consistent in nerve transposition repair [42]. The conventional suturing approach is to suture the epineurium, namely, the epineurial suture. When applying the epineurial suture to repair nerves with significant diameter disparities, potential tension at the suture site may arise. The suture site may develop neuromas as a result, causing nerve pain after repair [43]. Moreover, the presence of local tension impedes the growth of the proximal nerve, thereby influencing nerve regeneration post-suturing [44]. The conduit suturing method with a small gap in this study solved this problem. By suturing the nerves at both ends with minimal tension, the occurrence of local neuromas and secondary neuralgia can be significantly reduced. Furthermore, the technique maintains the gaps between nerve stumps, and research has indicated that the regrowth of axons is associated with the distance separating the nerve stumps [45,46]. In previous studies, we found that nerve regeneration was relatively better when the regeneration gap in rat peripheral nerves was 1–2 mm [8,47]. In this study, the gap was 2 mm, which neither affected nerve regeneration nor the accuracy of nerve regeneration.

When repairing large nerves with small nerves using conduits that have the same inner diameter at both ends, several issues may arise. If the inner diameter of the conduit matches the proximal donor nerve, compression on the distal recipient nerve can occur. Conversely, if the inner diameter of the conduit matches the distal recipient nerve, there may be an excessive gap between the inner wall of the conduit and the proximal donor nerve, leading to the potential escape of more regenerated nerve fibers and an increased likelihood of neuroma formation. Hence, this study effectively addressed this issue by employing conduits of varying inner diameters at both ends. The inner diameter of the narrower end of the conduit is 0.8 mm, which matches the diameter of the proximal common peroneal nerve. The inner diameter of the wider end of the conduit is 1.6 mm, making it appropriate for the diameters of the distal tibial nerve. The conduit’s inner diameter is slightly larger than the nerve’s diameter with a range of 0.1 to 0.2 mm due to three primary factors. First, inserting the nerve into the conduit and performing suturing without tension is a simple task. Additionally, following nerve injury, there will be tissue swelling, which leads to an increase in nerve diameter, allowing it to fit perfectly with the conduit’s diameter. This prevents any obstruction of blood supply caused by the compression of nerve tissue. Additionally, by populating the conduits with nerves, the leakage of regenerated nerve fibers will be minimized, effectively preventing the development of neuromas.

Chitosan served as the primary component for fabricating nerve conduits in this investigation. Chitosan exhibits numerous outstanding characteristics, including biocompatibility, biodegradability, antimicrobial attributes, and multifunctionality, which render it a highly promising substance within the realm of biomedicine [15]. The utilization of chitosan-based conduit during the mending process of injuries to peripheral nerves facilitates the smooth transition of regenerated nerve fibers toward the distant nerve, ultimately leading to a triumphant restoration. The initial step of this research involved dissolving chitosan in acetic acid solution to create a chitosan solution. Then, the solution is evenly covered on the surface of the customized mold. The mold consists of two cylindrical guiding rods with varying diameters that are connected by a conical piece. The diameter of the thinner guiding rod is 0.8 mm, and the diameter of the thicker guiding rod is 1.6 mm. The action of alkaline sodium hydroxide causes a change in the pH of the acidic chitosan solution, which in turn transforms it into a solid form. Then, chitosan is acetylated to form chitin using acetic anhydride, resulting in a reduction in conduit hardness and an increase in elasticity. This method is not only easy to operate but can also customize conduits of different specifications according to different conditions of nerve injury. At 16 weeks after surgery, in the CT, CT/GM, and CT/GM/GF groups, no obvious inflammatory reaction was observed at the nerve suture site. The conduits showed partial absorption, and there was no obvious local neuroma.

Although the utilization of conduits featuring different sizes at each end for nerve transposition repair offers numerous advantages, it solely tackles the issue of surgical suturing, indicating the potential for enhancing the repair outcome. The regeneration of peripheral nerves encompasses an intricate sequence of occurrences, which comprise sprouting of axons, reformation of myelin, and innervation of the target. The process of axonal sprouting involves the development of fresh axons originating from the nearby end of a damaged nerve, elongating along Schwann cell pathways and reaching the far end of the nerve. After reaching the distant stump, axons initiate the process of remyelination. The reestablishment of the functional connection between the regenerating axon and its target organ is the ultimate goal in the process of regeneration, which occurs during target innervation [21,48]. In general, growth factors play a vital role in the healing of peripheral nerve damage by promoting the growth, development, and survival of nerve cells, ultimately leading to improved nerve regeneration and functional recovery. VEGF is a crucial protein involved in promoting the growth of blood vessels and plays a significant role in the regeneration of nerves. VEGF facilitates nerve regeneration through various complex mechanisms. First, it stimulates the growth of new blood vessels, which help deliver nutrients and oxygen to regenerating nerve tissue. Furthermore, it facilitates the preservation and development of nerve cells through the prevention of cell death and the encouragement of axon extension. Ultimately, it boosts the development of the extracellular matrix, creating a conducive environment for the regeneration of nerves [22,49]. NGF is a protein synthesized by various cells in the body, such as Schwann cells, vascular endothelial cells, and macrophages, to enhance the development and viability of nerve cells. NGF enhances the viability and differentiation of neurons while also facilitating axon growth and providing guidance toward target cells. Additionally, it promotes the formation of fresh synapses and plays a crucial role in the restoration of peripheral nerves [50]. Hence, VEGF and NGF hold great potential as targets for the advancement of therapeutic strategies aimed at enhancing neural restoration. In addition, VEGF and NGF may synergistically promote nerve regeneration by facilitating angiogenesis and enhancing blood flow. VEGF stimulates angiogenesis and increases oxygen and nutrient supply, including NGF, to the injured area. Moreover, NGF upregulates VEGF expression, further augmenting angiogenesis. Consequently, VEGF and NGF exhibit complementary effects fostering nerve regeneration. The combination of their administration presents a hopeful approach to improve the regeneration and functionality of nerves [24]. Previous findings have shown that the combined application of VEGF and NGF can promote the repair of nerve damage [25].

To facilitate the healing of peripheral nerve damage using growth factors, a crucial issue is how to ensure the prolonged effectiveness of the medication at the site of the injury. Hydrogels are gel materials with physical or chemical cross-linking, affording them customizable physical and chemical characteristics. Their three-dimensional water-filled network structure fosters excellent compatibility between hydrogels and organisms [51]. In addition to their inherent therapeutic capabilities, hydrogels can serve as carriers for pharmacological agents, enabling sustained release within the body and enhancing drug stability [52]. Therefore, hydrogels have unique attributes that make them attractive as drug delivery platforms. GM, as a widely used hydrogel material, combines the favorable characteristics of both natural and artificial biomaterials [53]. GM demonstrates outstanding biocompatibility and cell growth, as well as a differentiation-friendly three-dimensional structure that stimulates cellular responses. Moreover, GM boasts adjustable mechanical properties, favorable degradability, and an internal porous structure that facilitates growth factor delivery. The remarkable biological properties of GM enable the preservation of growth factor activity during the delivery process, offering a foundation for tissue repair [54]. Consequently, GM has found extensive applications in the development of growth factor delivery systems.

In this study, we prepared GM hydrogels that contained VEGF and NGF. The hydrogels containing VEGF and NGF were characterized by their loose and porous structure, enabling the gradual release of growth factors. In vitro cytological experiments demonstrated that hydrogels promoted the proliferation, tube formation, and migration of HUVECs while decreasing apoptosis in PC-12 cells. Subsequent animal experiments in rats revealed that the simultaneous utilization of hydrogels containing two growth factors and chitin conduits possessing different inner diameters on both ends considerably amplified the motor function of nerves and the restoration of the nerve conduction function within a span of 16 weeks. Histological analysis confirmed enhanced nerve regeneration, the restoration of gastrocnemius muscles, and neuronal recovery. These findings confirm the effectiveness of using dual growth factor hydrogels in combination with chitin conduits for nerve transposition repair.

An interesting finding was observed in this study. In the in vitro trial, no notable disparity was found in the cellular functionality between the injury/GM group and the injury group or between the GM group and the control group. Nevertheless, during the in vivo trial, the nerve regeneration of the CT/GM group exhibited a certain degree of improvement compared to that of the CT group. One potential explanation for this finding is that the GM hydrogel was not loaded with growth factors, which had no notable impact on the in vitro proliferation, migration, and tube formation of HUVECs. Additionally, it had no significant effect on the apoptosis of PC-12 cells. Nevertheless, due to the similarity between the hydrogel to the extracellular matrix, it facilitates the attachment of cells in the affected region, potentially aiding in the restoration of nerve injury. As a result, this promotes nerve regeneration and enhances functional recovery in the in vivo experiments. Importantly, the original function of the proximal and distal nerves in nerve transposition repair is not the same. While regenerated nerve fibers can reach target organs for nerve regeneration, further neural functional remodeling is required for complete nerve repair. This process is time-consuming and critical for clinical nerve transposition repair.

This preliminary study demonstrates the therapeutic potential of utilizing hydrogels containing dual growth factors in conjunction with conduits of varying inner diameters at both ends for peripheral nerve injuries. However, further research is warranted to advance this approach. Future research will focus on developing novel models for nerve transposition repair and designing conduits that can better accommodate the diverse types of nerve injuries. Additionally, efforts will be directed toward enhancing hydrogel materials, improving growth factor loading strategies, and aligning the release kinetics of the growth factors with the requirements of the reparative microenvironment, leading to an improved repair of the nerve damage outcomes. Moreover, the underlying molecular mechanisms of the combined application of dual growth factor hydrogels and conduits with different inner diameters will be elucidated. Finally, it is important to note the evaluation of the repair efficacy was limited to a 16-week follow-up period, and an investigation into the long-term results is necessary.

## 5. Conclusions

In conclusion, the application of chitosan-prepared chitin conduits featuring varying inner diameters at both ends, together with GM hydrogels containing dual growth factors (VEGF and NGF), considerably enhances nerve regeneration and functional recovery following damage. As a result, the effectiveness of nerve transposition repair is improved. This approach holds significant potential in the clinical treatment of severe peripheral nerve injuries and contributes to advancing more effective treatments.

## Data Availability

The data are available from the corresponding author upon reasonable request.
